# Pathways to “Evidence-Informed” Policy and Practice: A Framework for Action

**DOI:** 10.1371/journal.pmed.0020166

**Published:** 2005-05-31

**Authors:** Shelley Bowen, Anthony B Zwi

## Abstract

Bowen and Zwi propose a new framework that can help researchers and policy makers to navigate the use of evidence.

The contemporary public health effort sees much debate about the concepts of “evidence” and “the evidence base”, and the usefulness and relevance of such terms to both policymaking and practice. A key challenge to public health is to better contextualize evidence for more effective policymaking and practice. Theory on the translation of research findings into policy and practice, and on knowledge utilization, offers only part of the solution to this complex task. The policymaking context is highly political and rapidly changing, and depends on a variety of factors, inputs, and relationships.

In this article, we propose that an “evidence-informed policy and practice pathway” can help both researchers and policy actors navigate the use of evidence (Figure 1). The pathway illustrates different types of evidence and their uses in health policymaking, and proposes that specific capacities, such as an individual's skills, experience, and participation in networks, influence the adoption and adaptation of evidence in practice.

**Figure 1:**
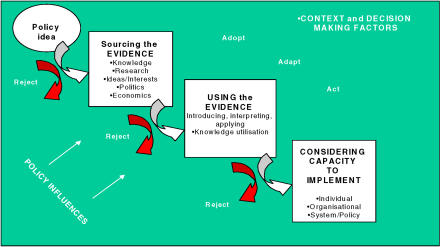
The Evidence-Informed Policy and Practice Pathway

The pathway to “evidence-informed” policy and practice involves three active stages of progression, influenced by the policy context. The three stages are (1) sourcing the evidence, (2) using the evidence, and (3) implementing the evidence. The pathway also involves decision-making factors and a process which we have termed “adopt, adapt, and act”. Once adopted, evidence about implementation is usually adapted or changed before use in the policy context. Policy actors and practitioners rightfully need to understand and decide how best this evidence should be acted upon in each circumstance. Each stage in this pathway is underpinned by a variety of individual-, organizational-, and system-level values.

To formulate the evidence-informed policy and practice pathway presented in this paper, we reviewed relevant literature from health, public policy, and the social sciences.

## Diffusion of Innovations: An Underlying Theory

Fundamental to the transfer of evidence into policy and practice is diffusion, the process by which an innovation is communicated over time among members of a social system (classical diffusion) [1]. In this paper we consider innovation to be the policy idea. Studies of innovation in health-care organizations by Lennarson Greer [2] proposed that diffusion theory helps us understand the following: (1) how individuals within an organization receive, adopt, and adapt evidence; (2) the organizational factors that constrain or facilitate the adoption or implementation of the evidence; and (3) the interests and values at play within organizations that influence responses to the evidence/policy issue.

The success of diffusion of evidence into policy and practice rests largely with the characteristics at play at each stage of the adoption process [3,4]. Information passes through an “adopt, adapt, and act” cycle. Characteristics of the individuals involved, the innovation itself, and the organizations in which they are considered affect decisions made about evidence in terms of the perceived value, priority given, and seriousness of response. The extent to which individual-, organizational-, and system-level values influence a decision to accept or reject the policy-related evidence is largely unexplored in the literature [5]. For example, the importance of values as a factor that influences the lack of action on health inequity has been poorly researched.

## Stage 1: Sourcing the Evidence

Evidence-informed policymaking sees the use of different types of information in a variety of forms and from a variety of sources, reflective of, and responsive to, the policy and practice context. Types of evidence that inform the policy process can be grouped as research, knowledge/information, ideas/interests, politics, and economics (see Table 1) [6]. Evidence is usually sought to show effectiveness (“it works”), show the need for policy action (“it solves a problem”), guide effective implementation (“it can be done”), and show cost effectiveness (“it is feasible and may even save money”).

**Table 1:**
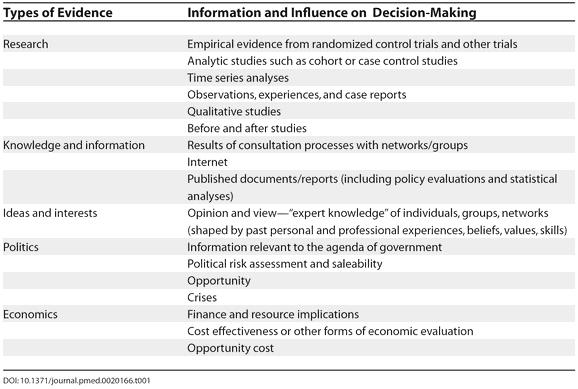
Types of Evidence and How They Are Used in Policy Making

The term evidence-based policy is used in the literature, yet largely relates to only one type of evidence—research. Using the term “evidence-influenced” or “evidence-informed” reflects the need to be context sensitive and consider use of the best available evidence when dealing with everyday circumstances [7–9]. A variety of distinct pieces of evidence and sources of knowledge inform policy, such as histories and experience, beliefs, values, competency/skills, legislation, politics and politicians, protocols, and research results [10,11]. Policy analysis theory proposes that evidence is information (information is data that has meaning) “that affects existing beliefs of important people about significant features of the problem under study and how it might be solved or mitigated” [12]. A case study of applying evidence to policy and practice in the real world is described in Box 1.

**Box 1. Case Study of Applying Evidence to Policy and Practice in the Real World**

The Kings Fund recently reviewed investments by the United Kingdom government into major social programmes. The Fund's report, called *Finding Out What Works*, asks to what extent these programmes are evidence based, what is being done to find out if they work, and whether the evaluations are helping to inform policy and practice in the future [54].

The report concludes that programmes like Sure Start (a government programme involving early interventions to improve children's social and educational welfare; see http://www.surestart.gov.uk) are largely driven by “informed guesswork, expert hunches, political and other imperatives”.

In this case, the application of evidence to policymaking was hindered by a lack of good-quality, synthesized evidence, capacity to apply the evidence, and organizational support and resources to make evidence-based decisions.

A real conflict exists between local control of decision-making and the idea of evidence-informed decision-making and evidence-based policy and practice. The report's authors state that “decisions are guided by common sense and experience rather than the formal evidence base”.

The way in which research evidence is combined with other forms of information is key to understanding the meaning and use of evidence in policy development and practice. Current literature on evidence-based health care is often limited by inadequate attention to context [4,13,14]. A major challenge to contextualizing evidence for policymaking is recognition that a broad information base is required [4].

## The Policy Context

Considering the evidence within the context in which it will be used is critical for effective policymaking and practice. The context is the environment or setting in which the policy is being developed and implemented [15], incorporating the historic, cultural, health services, system, and resource contexts. The social and political context and the many forces at work in the policy environment provide challenges to integrating evidence into policy and practice. Researchers often do not see or recognize these factors. The political, ideological, and economic factors influencing policy development and decision-making often gain strength at the expense of the research evidence; a recent example is the political commitment in Australia to establish a range of new medical schools rather than enable existing schools to train more people, which would likely be far more cost-effective.

The ways in which the evidence is used in the policy process are largely determined by the beliefs and values of policymakers, as well as by considerations of timing, economic costs, and politics [2,18–22]. The development of both the Black report [16] and the *Independent Inquiry into Inequalities in Health Report* in England [17] are illustrative. The Black report was dismissed by the Conservative government of the time despite extensive evidence on inequality. Almost 20 years later the *Independent Inquiry into Inequalities in Health Report* and subsequent policy actions were largely driven by government. How and when evidence is used often depends upon the political agenda and ideology of the government of the day, not on the nature of the evidence, however compelling [20,23].

Policy networks provide a useful lens through which to analyze the context of policymaking and research utilization. A policy network focuses on the relationships that shape the policy agenda and decision-making process. Networks can shape the way policy is formulated, and in particular the way in which evidence is gathered and presented in policy formulation [24,25]. Epistemic communities are formal and informal groups of technical “experts” who purvey information and share ideas about research data, knowledge, and experience. These communities gather, synthesize, and disseminate information about a policy issue, as well as advocate for knowledge transfer across social systems and government [26,27].

## Factors in Decision-Making

### The usefulness of the innovation

Decisions about the usefulness of an innovation itself are often based on relative advantage (is the innovation better than previous approach?); complexity (is the innovation understandable?); compatibility with values and past experiences; and cost and flexibility, reversibility, trialability, and revisability (is there opportunity to trial and change?) [1,2,28–30]. Potential adopters who see the innovation as compatible with their values and those of their organizations are more likely to adopt than those who do not [3]. The literature also suggests that organizations that are close to each other—geographically or in communication—will adopt innovations because of the “bandwagon effect” [3,31].

The rapid diffusion of new hospital equipment [32] versus the slow diffusion of ideas on sudden infant death syndrome [33] or policy to tackle health inequalities in health services [34] provide examples of how different ideas and forms of knowledge on different issues can result in different diffusion efforts and successes.

### The influence of the individual

Individuals are key participants in decisions about use of evidence throughout the policy and practice pathway, as it is individuals who decide whether to accept or reject something new. Individual decisions are influenced by a variety of personal qualities and capacities such as values and beliefs, leadership, knowledge and skills, resources, organizational support, partnership links, and networking. Additionally, individuals are influenced by the perceived benefit of change, and, once again, by the complexity of the innovation itself. Individuals often avoid change, reinforcing organizational inertia [32].

Classical diffusion theory identifies and categorizes adopters. Early adopters are defined as venturesome innovators, active seekers of new ideas, favourable to change, willing to take risks, part of a highly interconnected social system and networks, and cosmopolitan [2,3]. The early adopters are deliberate, the late majority sceptical, and the belated adopters traditional [1]. Late adopters are often influenced most strongly by local experience and interpersonal contact. Greenhalgh and colleagues [35] advocate that diffusion is influenced more by broader organizational and environmental factors and less by an individual's adoption style [35].

### The influence of the organization.

In health systems, groups of individuals, the structure of the organization they are a part of, and the broader policy context influence decision-making and the diffusion of ideas [32]. An organization's structure, function, composition, and socioeconomic context are primary influences on both what decisions are made and how they are made [2,32,36]. As an example, centralization or formalization of decision-making processes can affect adoption, affecting information flow. Organizational composition, the nature of staff, and the degree of skills and training can have a direct relationship to acceptance and change [36].

The extent to which change, new concepts, and new ideas are valued by management and leadership figures influences rates of adoption and adaptation [2,36]. Dealing with and accepting change, such as using research evidence in practice, calls for application of change theory, which proposes ideas, adoption, and implementation stages [2]. The ideas stage calls for flexibility and creativity, the adoption stage focuses on motivation, resource allocation, and negotiation, and the implementation stage is based on perceptions of legitimacy and an environment of trust [2,30,37,38].

## Stage 2: Using Evidence in Policymaking

A number of studies have considered the social and political environment in which evidence is used in policymaking, offering a series of models starting with problem identification through to collaborative interpretation, solution, and application [39–41]. Staged models, whilst insightful, can suggest that policymaking is a logical, rational, and linear process. It is difficult for evidence to remain intact through the process given the policy context, decision-making factors, and the need to adapt. This indicates two things, that the evidence interacts with “context” before it is fully adopted in policy and practice, and/or that different types of evidence are useful at different times in the policy process.

The literature identifies at least three key stages of knowledge utilization: introduction, interpretation, and application [39–42]. Table 2 suggests a variety of considerations as research evidence passes through three stages of use, sourced from the work of Dobrow and colleagues [40].

**Table 2:**
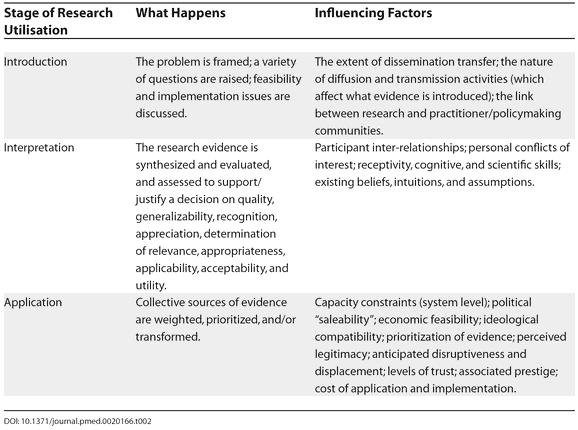
Stages of Research Utilization

Effective knowledge transfer is not a “one off” event, rather it is a powerful and continuous process in which knowledge accumulates and influences thinking over time [43]. The ability to sustain this process and a focus on human interactions is essential [43,44]. Differences in conceptual understanding, scientific uncertainty, timing, and confusion influence the response to evidence. There is no shortage of great ideas presented to policymakers in which the evidence might be either insufficient or overrepresented, often leading governments into decision-making with inadequate information [4,23].

Understanding knowledge utilization in policymaking requires an understanding of what drives policy. A variety of policy processes may be operating that influence the climate for accepting different types of evidence. As proposed by Weiss in the late 1970s [45], policy models influence where, when, and if evidence is used. A combination of the models presented in Box 2 best describes the policymaking process.

**Box 2. Policymaking Models and the Use of Research Evidence**

**The Knowledge-Driven Model:** This model suggests that emergent research about a social problem will lead to direct application to policy; it relies on effective strategies for the transfer of research evidence into practice.

**The Problem-Solving Model:** This model expects research to provide empirical evidence and conclusions that help solve a policy problem; it assumes that evidence is systematically gathered and applied in the policy process.

**The Interactive Model:** This model suggests that the search for knowledge moves beyond research to include a variety of sources such as politics and interests; it aims to reflect the complexity of the policymaking process.

**The Political Model:** In this model, decision-makers are not receptive to research unless it serves political gain, that is, demonstrates proof for a predetermined decision; evidence is sought to justify the problem.

**The Enlightenment Model:** This model suggests that cumulative research shapes concepts and perspectives that permeate the policy process over time, influencing how people think about social issues.

**The Tactical Model:** This model sees evidence used to support and justify government inaction, or rejection of and delay in commitment to a policy issue [23,45].

## Stage 3: Capacity for Implementation

Determining capacity to act on evidence is a neglected area of policy analysis and research efforts to date. This gap exists largely because capacity is a difficult concept to define and subsequently to assess or measure [46–49]. Capacity in the health sector refers to the ability to carry out stated objectives; it is the expertise and resources at individual, organizational, and system levels for the production and application of new knowledge to health problems [50,51]. At the individual and organizational levels, capacity is often visible as skills and competencies, leadership, partnerships, the development of appropriate workforce and organizational structures, and the ability to mobilize and allocate resources [52,53]. Key at a system level are processes, policies, politics, and people (see Table 3). A case study showing how capacity is required to implement an idea informed by evidence is shown in Box 3.

**Box 3. Case Study Showing How Capacity Is Required to Implement an Idea Informed by Evidence**

McKee and colleagues [33] examined the diffusion of strategies for preventing sudden infant death syndrome internationally. Their analysis provides an example of a policy idea well supported by evidence, values, and cost effectiveness data, but poorly implemented. A few simple strategies have been found to reduce the risk of the syndrome, including sleeping in the supine position, avoiding exposure to tobacco smoke, breast feeding where possible, and avoiding overheating.

The authors found that although evidence about the role of sleeping position began to become available in the early 1980s, it was several years before it was acted upon, initially in the Netherlands and subsequently in New Zealand, the United Kingdom, and Scandinavia. Several countries have mounted major national preventive campaigns, but others have not. This case highlights the importance of considering implementation and systems/policy factors alongside the strength of the evidence.

**Table 3:**
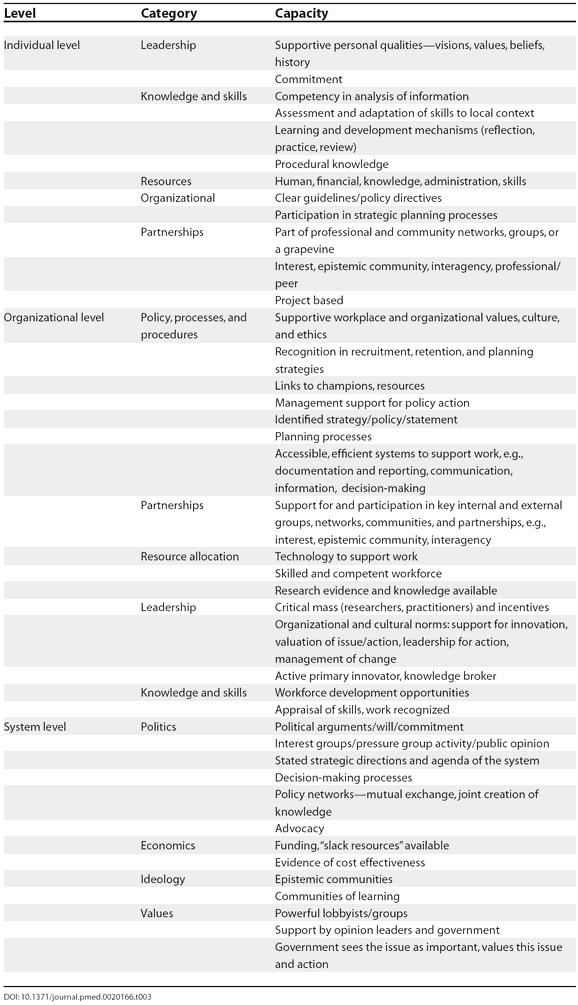
Capacities Required for Policy Adoption and Adaptation

The literature on capacity and capacity building adds value to what is already known about mechanisms for optimizing conditions for integrating research with policy and practice. Capacity theory offers something practical and operational, and calls for capacity to “adopt, adapt, and act” on the evidence in informing policy issues, otherwise policy remains idle [33,34,51]. This literature offers a more concentrated focus on individual-, organization-, and system-level factors as key to adoption, adaptation, and action in both developing and implementing evidence-informed policy. Capacity thinking both asks and answers the question of what needs to be in place to support evidence uptake in policy and practice across a variety of settings.

## Why This Framework?

The purpose of this framework is to describe the myriad of changing influences in achieving evidence-informed policy and practice. The framework encourages research and planning in the area of how to “adopt, adapt, and act” on the evidence and in capacity for implementation as part of the evidence-informed policy development process. The visual presentation and descriptive mapping of the stages and features offers opportunity for deepening our understanding of the connectedness (or non-connectedness) between these factors. It also helps identify potential interventions. The framework emphasizes the policy context and its influence on each stage of interaction between research, other forms of evidence, and the policy process. Defining different types of evidence helps to both value and guide the sourcing of a broad range of information for policymaking.

## Conclusion

Understanding how evidence informs policy and practice is critical in promoting effective and sustained public health action. The debate on evidence in public health has largely focussed on the linear use of research evidence in a programmatic rather than policy context. The starting point for navigating the use of evidence in policy and practice is understanding diffusion (how ideas spread throughout systems), how decisions are made, how policy is developed, and how capacity is required to effectively use evidence in this process.
